# Alignment error modeling and control of a double-sided microlens array during precision glass molding

**DOI:** 10.1038/s41378-024-00668-7

**Published:** 2024-04-07

**Authors:** Zihao Zeng, Tianfeng Zhou, Qian Yu, Jia Zhou, Gang Wang, Qiuchen Xie, Zifan Wang, Xiaoqiang Yao, Yubing Guo

**Affiliations:** 1 School of Medical Technology, Beijing Institute of Technology, No. 5 Zhongguancun South Street, Haidian District, Beijing, 100081 China; 2 School of Mechanical Engineering, Beijing Institute of Technology, No. 5 Zhongguancun South Street, Haidian District, Beijing, 100081 China

**Keywords:** Engineering, Optics and photonics

## Abstract

Double-sided microlens arrays (DSMLAs) include combinations of two single-sided MLAs to overcome positioning errors and greatly improve light transmissivity compared to other types of lenses. Precision glass molding (PGM) is used to fabricate DSMLAs, but controlling alignment errors during this process is challenging. In this paper, a mold assembly was manufactured with a novel combination of materials to improve the alignment accuracy of mold cores during PGM by using the nonlinear thermal expansion characteristics of the various materials to improve the DSMLA alignment accuracy. By establishing a mathematical model of the DSMLA alignment error and a thermal expansion model of the mold-sleeve pair, the relationship between the maximum alignment error of the DSMLA and the mold-sleeve gap was determined. This research provides a method to optimize the mold-sleeve gap and minimize the alignment error of the DSMLA. The measured DSMLA alignment error was 10.56 μm, which is similar to the predicted maximum alignment error. Optical measurements showed that the uniformity of the homogenized beam spot was 97.81%, and the effective homogeneous area accounted for 91.66% of the total area. This proposed method provides a novel strategy to improve the performance of DSMLAs.

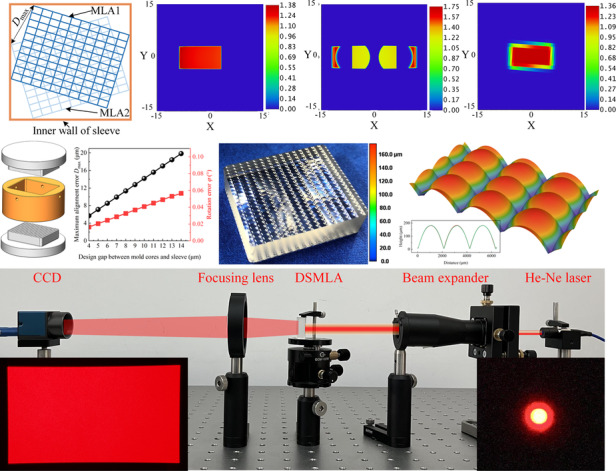

## Introduction

Double-sided microlens arrays (DSMLAs) work to homogenize laser beams by dividing the incident nonuniform beam into multiple beams and superimposing these beams using a focusing lens. DSMLAs exhibit simple structures, low transmission losses, high damage thresholds, and low requirements for incident light intensity distribution, making them widely used for laser beam homogenization^1,2^ and structured illumination^3,4^. Furthermore, DSMLAs are aligned with the development direction of microelectromechanical systems (MEMSs) due to their attributes of miniaturization, integration, and light weight. As a result, the DSMLA has been extensively utilized in MEMS applications such as laser projection^5^, laser manufacturing^6^, and lithography^7^. Commonly used processing methods for microlens arrays include lithography^8,9^, microplastic embossing^10^, and thermal reflow technology^11^, but material limitations result in polymer microlens arrays with limited unit apertures. Laser direct writing^12,13^ is also widely used to fabricate microlens arrays. While this method is suitable for the production of single-sided glass microlens arrays, ensuring high alignment accuracy and sufficient surface quality are challenging for double-sided glass microlens arrays. The optical performance of a DSMLA largely depends on the alignment error incurred during manufacturing of both sides of the microlens array (MLA)^14,15^. Various approaches have been used to fabricate DSMLAs with high alignment accuracy. Yang et al. fabricated a DSMLA with a limited focal range using a thermal reflow technique^3^. Xue et al. used photolithography to fabricate discontinuous light-cured polymer biconvex MLAs on both sides of glass lenses and used collimated light to reduce the alignment error of the DSMLA^16^. Cheng et al. fabricated two MLA molds by slow tool servo machining and then used injection molding to prepare a DSMLA made of PMMA, including the use of positioning pins to ensure DSMLA alignment accuracy^17^. The lenses fabricated by the above methods were pure-polymer DSMLAs or a polymer was used to fabricate a DSMLA on a glass surface. The use of polymers resulted in shorter lifetimes and worse high-temperature resistance than did conventional pure-glass MLAs.

Precision glass molding (PGM) replicates the surface morphology of a mold core on a glass surface by heating the glass until it softens, after which it is deformed by precisely applying pressure^18,19^. The high-precision mold core ensures high profile accuracy and surface quality of the MLA. Glass MLAs manufactured by this method exhibit consistent lens units, high production efficiencies, and low costs^20,21^. Most of those previous studies focused on the PGM of single-sided glass MLAs with high forming quality^22,23^, but few researchers have attempted to fabricate DSMLA by PGM. Huang et al. used a laser to fabricate silicon carbide mold cores with microporous arrays and subsequently used them to fabricate a DSMLA by PGM^24^. Zhang et al. used single-pulse femtosecond laser-assisted chemical etching to directly manufacture a concave lens array mold of glass material and then manufactured an infrared DSMLA by PGM^25^. However, the alignment error of the DSMLA was not fully considered in these studies, such that a suitable strategy to predict and correct the alignment error of the DSMLA remains to be demonstrated.

In this work, we report such a strategy for fabricating a large-area and high-precision DSMLA by designing molds with different materials for use in PGM. First, we analyzed the influence of alignment error on the beam homogenization effect of the DSMLA and studied the relationship between the mold design gap and the maximum alignment error of the DSMLA. Next, a nonlinear thermal expansion mathematical model characterizing the mold core and sleeve was derived, and the mold was designed accordingly. Finally, the feasibility of this method for fabricating a DSMLA with high alignment accuracy was experimentally verified, and the optical performance of the fabricated DSMLA was evaluated using a beam-homogenizing optical circuit.

## Description and modeling of DSMLA alignment error

### Description of the DSMLA alignment error

**Fig. 1:**
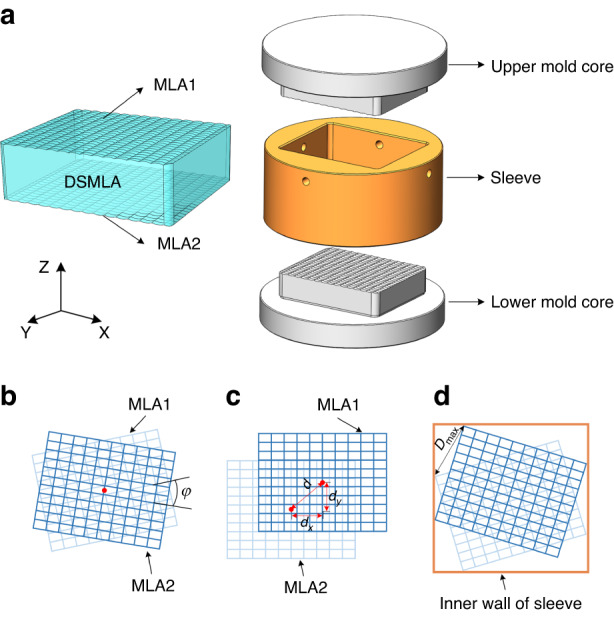
The causes of DSMLA alignment error during the PGM. **a** DSMLA and the mold assembly in PGM. **b** Rotation error, **c** shift error *d* including the shift error *d*_x_ and shift error *d*_y_, and **d** alignment error of the DSMLA

As shown in Fig. 1a, the mold for the MLA PGM comprises an upper mold core, a lower mold core, and a sleeve. During the molding process of the DSMLA, the gap between the mold cores and sleeve at high temperatures affects the alignment error of the upper and lower mold cores along the *x–y* plane.

The DSMLA included two sides: MLA1 and MLA2. Possible alignment errors of the DSMLA are shown in Fig. 1b–d, including rotation error and shift error. When MLA1 and MLA2 rotated relative to the optical *Z* axis, the rotation angle *φ* was denoted the rotation error. When MLA1 and MLA2 shifted relative to each other in the *x*- and *y*-directions, the translation distance *d* was denoted the shift error. The rotation error and the shift error affected the alignment error of the DSMLA. The alignment errors of each lens unit in MLA1 and MLA2 were different, and the maximum alignment error of the lens unit in the DSMLA was denoted *D*_max_.

The mold cores and sleeve are typically made of the same material to maintain consistency of the gap at different temperatures, thus ensuring the accuracy of the alignment of the DSMLA at high temperatures. Commonly used mold core materials for PGM include cemented carbide^26,27^ and heat-resistant stainless steel with nickel-phosphorus (Ni-P) plating^28,29^.

The thermal expansion coefficient of cemented carbide is less than that of glass, and fabricating a sleeve of this material can simplify the demolding process. However, the poor processability of cemented carbide makes this material difficult to use for fabricating large-area high-precision MLA mold cores^30,31^. Ni-P plating on heat-resistant stainless steel can be used to fabricate high-precision MLA mold cores^32,33^. However, the thermal expansion coefficient of heat-resistant stainless steel is greater than that of glass, which prevents the glass MLA from releasing from the sleeve after PGM in cases where the mold cores and sleeve are made of heat-resistant stainless steel.

In general, achieving high alignment accuracy for DSMLAs, smooth demolding of lenses, and high surface accuracy for large-area manufacturing of MLA are challenging when the mold cores and sleeve are made of the same material. Here, we propose the use of different materials to fabricate mold cores and sleeves to obtain a high-performance DSMLA. Optical glass molding is typically conducted at temperatures ranging from 400 to 600 °C^34,35^, at which the mold cores and sleeves undergo different degrees of thermal expansion, causing the gap between them to change significantly. Therefore, the intended gap based on different material cores and sleeves under room temperature conditions differs from the gap occurring at higher temperatures. As yet, there is no design theory or mature method for fabricating multi-material molds for DSMLAs and overcoming this temperature effect.

### Impacts of DSMLA alignment error

**Fig. 2:**
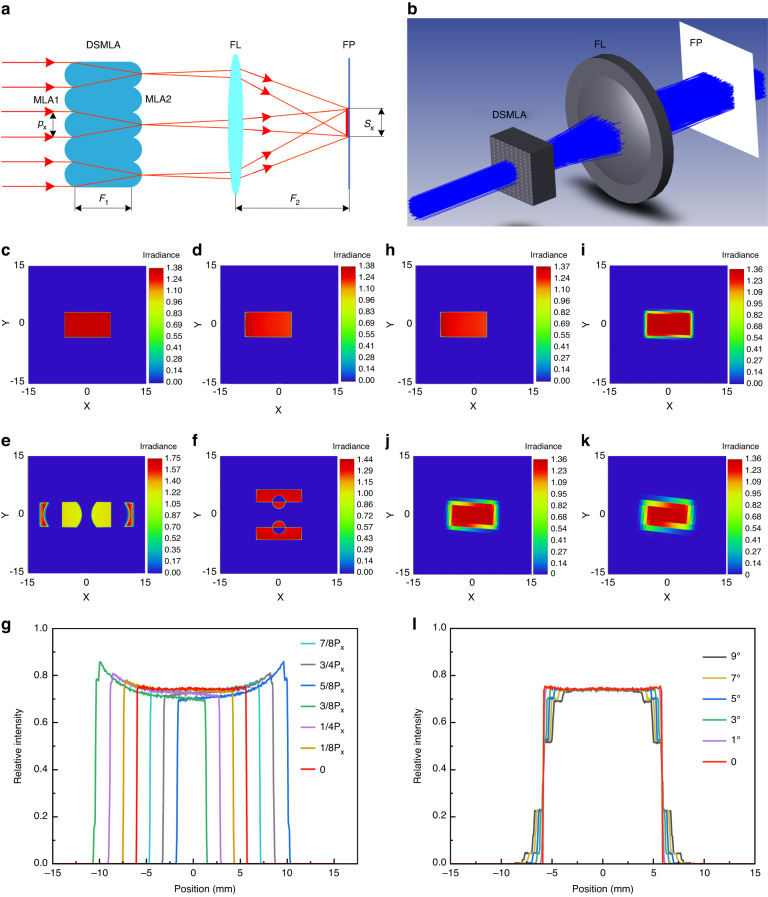
The influence of DSMLA alignment error on the beam homogenization effect. **a** Diagram of the principle of beam homogenization based on a DSMLA. **b** Optical simulation model of beam homogenization based on a DSMLA. Spot uniformity under different shift errors *d* of the DSMLA: **c** *d*_x_ = 0, **d** *d*_x_ = 1/4*p*_x_, **e** *d*_x_ = 1/2*p*_x_, and **f** *d*_y_ = 1/2*p*_y_. **g** Variation in the light intensity. Spot uniformity under different rotation errors *φ* of the DSMLA: **h** *φ* = 1°, **i** *φ* = 3°, **j** *φ* = 5°, and **k** *φ* = 7°. **l** Variations in the light intensity

The principle of the uniform optical path composed of a DSMLA and a focusing lens (FL) is shown in Fig. 2a. The thickness of lens *F*_1_ was consistent with the focal length *f*_1_ of MLA1. The incident beam was divided into multiple beams by MLA1, focused at MLA2, and then overlapped on the imaging plane FP by a focusing lens with a focal length of *F*_2_. Accordingly, a homogenized beam spot was obtained.

The incident light was modeled as a collimated Gaussian beam, and the size *S*_x_ in the *x*-direction of the homogenized spot was defined by Eq. (1)^36^:

1 $${S}_{x}=2{F}_{2}\,\tan \left(\arcsin \left[\frac{n}{\sqrt{1+{(2{F}_{1}/{p}_{{\rm{x}}})}^{2}}}\right]\right)=\frac{2{F}_{2}n}{\sqrt{1+{(2h/{p}_{x})}^{2}-{n}^{2}}}$$

where *n* is the refractive index of the glass and the size of the lens unit is *p*_x_•*p*_y_ (*p*_y_ < *p*_x_).

Based on the above beam homogenization principle, the Zemax simulation model shown in Fig. 2b was established to analyze the influence of alignment error on beam homogenization. The shift error of the DSMLA along the *x*-direction was *d*_x_, and the shift error along the *y*-direction was *d*_y_. Both errors had similar effects on the beam spot, so the shift error *d*_x_ was taken as an example for analysis.

The relationship between the spot uniformity and shift error is shown in Fig. 2c–g. When *d*_x_ increased from 0 to 1/2*p*_x_, the spot uniformity gradually decreased, and the spot center shifted in the *x*-direction. When *d*_x_ = 1/2*p*_x_, the spot fails, as shown in Fig. 2e; when *d*_y_ = 1/2*p*_y_, the spot also fails, as shown in Fig. 2f. When *d*_x_ increased from 1/2*p*_x_ to *p*_x_, the spot uniformity gradually increased, and the spot center gradually returned to its initial position. When *d*_x_ = *p*_x_, the spot was consistent with its initial state.

The relationship between the spot uniformity and rotation error *φ* of the DSMLA is shown in Fig. 2h–l. As *φ* increases, the overall area of the spot increases, the area of the central uniform area decreases, and the sharpness of the spot edge decreases, while the central light intensity remains unchanged. These simulation results suggest that the shift error *d* and rotation error *φ* of the DSMLA reduce the spot uniformity.

### Modeling of DSMLA alignment error

**Fig. 3:**
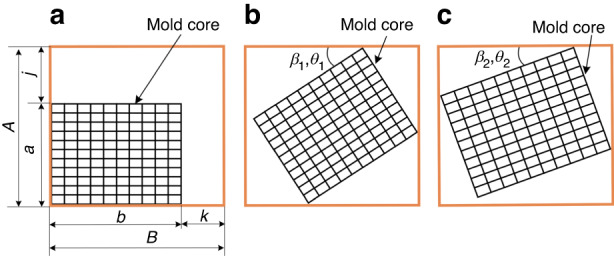
DSMLA maximum alignment error model. **a** The gap between the mold cores and the sleeve. Maximum rotation angle of the mold cores in the sleeve: **b** *β*_1_ and *ϴ*_1_ and **c** *β*_2_ and *ϴ*_2_

The PGM process includes four stages^18^: heating, molding, annealing, and cooling. During the molding stage, the gap between the mold cores and the sleeve determines the theoretical maximum alignment error of the DSMLA, which is determined by establishing a mathematical model describing the maximum gap between the mold cores and the sleeve. As shown in Fig. 3, the sleeve and the mold cores were designed with a clearance fit. The inner wall size of the sleeve was set to *A* × *B* (*A* < *B*). The overall size of the upper mold core was *a*_1 _× *b*_1_ (*a*_1_ < *b*_1_), the overall size of the lower mold core was *a*_2_ × *b*_2_ (*a*_2_ < *b*_2_), and the gaps between the mold cores and the sleeve were *k* and *j*, respectively.

Due to the uncertainty of the size of the mold cores and sleeve, the maximum relative rotation angle of the upper and lower mold cores was established according to two scenarios. When the upper mold core rotates and first contacts the sidewall of the sleeve with side length *B*, the relative rotation angle of the upper mold core is *β*_1_. When the upper mold core rotates and first contacts the sidewall of the sleeve with side length *A*, the relative rotation angle of the upper mold core is *β*_2_. The minimum values of *β*_1_ and *β*_2_ are the maximum rotation angle *β* of the upper mold core in the sleeve.

When the lower mold core rotates and first contacts the sidewall of the sleeve with side length *B*, the relative rotation angle of the lower mold core is *ϴ*_1_. When the lower mold core rotates and first contacts the sidewall of the sleeve with side length *A*, the relative rotation angle of the lower mold core is *ϴ*_2_. The minimum values of *ϴ*_1_ and *ϴ*_2_ are the maximum rotation angle *ϴ* of the lower mold core in the sleeve.

According to the limiting scenario, during the molding stage, the maximum alignment error occurs when the mold core rotates and there are three vertices in contact with the sleeve. At this time, *β*_1_ and *β*_2_ are defined by Eqs. (2) and (3), and *ϴ*_1_ and *ϴ*_2_ are defined by Eqs. (5) and (6), respectively. The maximum alignment errors of the upper mold core and lower mold core are divided according to two scenarios, which are defined by Eqs. (8) and (9), respectively:

2 $${{\rm{\beta }}}_{1}=90^\circ -\arctan \frac{{a}_{1}}{{b}_{1}}-\arccos \frac{{\rm{A}}}{\sqrt{{{{\rm{a}}}_{1}}^{2}+{{{\rm{b}}}_{1}}^{2}}}$$

3 $${{\rm{\beta }}}_{2}=90^\circ -\arccos \frac{{\rm{B}}}{\sqrt{{{{\rm{a}}}_{1}}^{2}{{+{\rm{b}}}_{1}}^{2}}}-\arctan \frac{{b}_{1}}{{a}_{1}}$$

4 $$\min ({\beta }_{1},{\beta }_{2})=\beta$$

5 $${{\rm{\theta }}}_{1}=90^\circ -\arctan \frac{{a}_{2}}{{b}_{2}}-\arccos \frac{{\rm{A}}}{\sqrt{{{{\rm{a}}}_{2}}^{2}+{{{\rm{b}}}_{2}}^{2}}}$$

6 $${{\rm{\theta }}}_{2}=90^\circ -\arccos \frac{{\rm{B}}}{\sqrt{{{{\rm{a}}}_{2}}^{2}{{+{\rm{b}}}_{2}}^{2}}}-\arctan \frac{{b}_{2}}{{a}_{2}}$$

7 $$\min ({\theta }_{1},{\theta }_{2})=\theta$$

8 $${D}_{\max 1}=\sqrt{{(B-{b}_{1}\cos {\rm{\beta }})}^{2}+{(A-{a}_{2}\cos {\rm{\theta }})}^{2}}$$

9 $${D}_{\max 2}=\sqrt{{(B-{b}_{2}\cos {\rm{\theta }})}^{2}+{(A-{a}_{1}\cos {\rm{\beta }})}^{2}}$$

The theoretical maximum alignment errors of the upper mold core and the lower mold core are the maximum values of *D*_max1_ and *D*_max2_, respectively, which are defined by Eq. (10). Because the alignment error is the same at both the molding temperature and room temperature, this can also be used to represent the maximum alignment error of the DSMLA at room temperature. The maximum rotation angle *φ* can be defined by Eq. (11):

10 $${D}_{\max }=\,\max ({D}_{\max 1},{D}_{\max 2})$$

11 $$\varphi =\beta +\theta$$

## Mold design and alignment error control

### Nonlinear thermal expansion model

Thermal deformation of the mold cores and sleeve at the molding temperature strongly influences the assembly accuracy. To control the gap between the mold cores and sleeve at high temperatures to ensure the alignment accuracy of the DSMLA, it is necessary to understand the thermal deformation of the mold cores at high temperatures and determine its impact on the mold assembly error. This approach ensures the high-precision fit of the mold cores and sleeve at molding temperatures. Therefore, the thermal expansion of sleeve and mold cores was theoretically deduced, and an accurate mathematical model of thermal expansion was established.

The thermal expansion coefficients of a material are divided into its linear thermal expansion coefficient and volume thermal expansion coefficient. The average linear expansion coefficient of a material is generally used in modeling. This linear expansion coefficient is related to the volume expansion coefficient of a material. To accurately calculate the change in size of a mold during thermal expansion and cooling, it is necessary to study the changes in its elastic modulus with temperature. As the temperature of the sample changed from *T*_1_ to *T*_2_, the length changed from *L*_1_ to *L*_2_ and the volume changed from *V*_1_ to *V*_2_. Moreover, *α*_L_ is the linear expansion coefficient, which can be defined by Eq. (12), and *α*_V_ is the volume expansion coefficient, which can be defined by Eq. (13)^37^. The relationship between the volume expansion coefficient and linear expansion coefficient is expressed by Eq. (14), where Δ*T* is the temperature change and the positive and negative Δ*T* values correspond to heating and cooling, respectively:

12 $${\alpha }_{{\rm{L}}}=\frac{({L}_{2}-{L}_{1})}{{L}_{1}({T}_{2}-{T}_{1})}$$

13 $${\alpha }_{{\rm{V}}}=\frac{({V}_{2}-{V}_{1})}{{V}_{1}({T}_{2}-{T}_{1})}$$

14 $${\alpha }_{{\rm{V}}}=3{\alpha }_{{\rm{L}}}\left(1+{\alpha }_{{\rm{L}}}\Delta T+\frac{{{\alpha }_{{\rm{L}}}}^{2}\Delta {T}^{2}}{3}\right)$$

The value of *α*_L_^2^Δ*T*^2^/3 is very small and can be neglected, such that Eq. (14) is simplified to Eq. (15):

15 $${\alpha }_{{\rm{V}}}=3{\alpha }_{{\rm{L}}}(1+{\alpha }_{{\rm{L}}}\Delta T)$$

where *α*_E_ is the coefficient of variation of Young’s modulus with temperature. According to thermoelastic theory, as the temperature increases, the thermal motion of atoms inside the metal increases, which increases the molecular spacing and decreases gravitational interactions between molecules, thereby increasing the ease of material deformation. The Young’s modulus *E* of a metal decreases linearly with increasing temperature, as shown in Eq. (16):

16 $$E={E}_{{\rm{T}}0}(1+{\alpha }_{{\rm{E}}}\Delta T)$$

where *E*_T0_ is the elastic modulus at room temperature, *α*_E_ < 0.

**Fig. 4:**
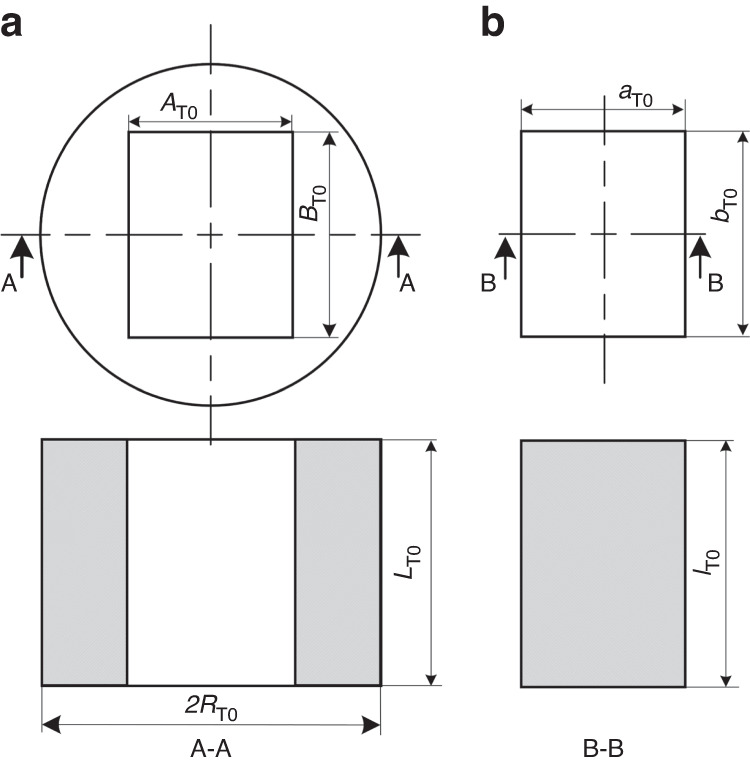
Simplified mold core and sleeve models. **a** Sleeve model at room temperature and **b** mold core model at room temperature

The sleeve is a hole-shaped part, as shown in Fig. 4a; its inner hole is rectangular and the outer wall of this hole is circular. The size of the sleeve’s inner wall at room temperature is *A*_T0_ × *B*_T0_, the radius of the sleeve’s outer wall at room temperature is *R*_T0_, the sleeve thickness is *L*_T0_, the density of the sleeve material is *ρ*, and the gravitational acceleration is *g*. The change in the size of the inner hole of the sleeve is determined as follows. The thermal deformation of the sleeve in the vertical direction (*Z*) is obtained as the sum of the deformation due to a change in its elastic modulus and the deformation caused by thermal expansion. The change in the overall height of the sleeve is represented by ∆*L*, which is defined by Eq. (17): ∆*L*_E_ is the deformation caused by changes in the elastic modulus, and *∆L*_α_ is the deformation caused by the thermal expansion of the material:

17 $$\Delta L=\Delta {L}_{{\rm{E}}}+\Delta {L}_{\alpha }$$

This deformation is caused by a change in the elastic modulus, which depends on the weight of the part. Considering a small segment *dh* from the bottom surface *h* of the part, the vertical load on *dh* is *F*_h_, which is defined by Eq. (18). The Young’s modulus *E* is defined by Eq. (19):

18 $${F}_{{\rm{h}}}=({L}_{{\rm{T}}0}-h)\cdot (\pi {{R}_{{\rm{T}}0}}^{2}-{A}_{{\rm{T}}0}{B}_{{\rm{T}}0})\rho g$$

19 $$E=\frac{{F}_{{\rm{h}}}/S}{dh/h}$$

The cross-sectional area is *S*, the elongation is *dL*, the length at room temperature is *L*_T0_, and the length after temperature change is *L*_T1_. ∆*L*_E_ is defined by Eq. (20):

20 $$\Delta {L}_{{\rm{E}}}={\int }_{0}^{{L}_{{\rm{T}}0}}\frac{({L}_{{\rm{T}}0}-h)\rho g}{{E}_{{\rm{T}}0}(1+{\alpha }_{{\rm{E}}}\Delta T)}dh=\frac{{{L}_{{\rm{T0}}}}^{2}\rho g}{2{E}_{{\rm{T0}}}(1+{\alpha }_{{\rm{E}}}\Delta T)}$$

Since *α*_E_ ≪ 1 and can be neglected, Eq. (20) is simplified into Eq. (21):

21 $$\Delta {L}_{{\rm{E}}}=\frac{{{L}_{{\rm{T0}}}}^{2}\rho g}{2{E}_{{\rm{T0}}}}\left(1-{\alpha }_{{\rm{E}}}\Delta T\right)$$

Thus, the ∆*L*_E_ includes two parts: ∆*L*_ET_, which changes with temperature, and ∆*L*_EG_, which is independent of temperature. Because ∆*L*_EG_ does not change before or after a temperature change, we consider only the partial change in height caused by temperature change, which is defined by Eq. (22):

22 $$\Delta {L}_{{\rm{ET}}}=-{\alpha }_{{\rm{E}}}\Delta T\frac{{{L}_{{\rm{T0}}}}^{2}\rho g}{2{E}_{{\rm{T0}}}}$$

The deformation caused by thermal expansion of the material is *∆L*_α_, which is defined as Eq. (22):

23 $$\Delta {L}_{\alpha }={L}_{{\rm{T0}}}{\alpha }_{{\rm{L}}}\Delta T$$

The height of the sleeve after thermal deformation is *L*_T1_, which is defined by Eq. (24):

24 $${L}_{{\rm{T1}}}={L}_{{\rm{T0}}}+\Delta {L}_{{\rm{ET}}}+\Delta {L}_{{\rm{\alpha }}}={L}_{{\rm{T0}}}-{\alpha }_{{\rm{E}}}\Delta T\frac{{{L}_{{\rm{T0}}}}^{2}\rho g}{2{E}_{{\rm{T0}}}}+{L}_{{\rm{T0}}}{\alpha }_{{\rm{L}}}\Delta T$$

The size of the outer wall of the sleeve after thermal deformation is *R*_T1_, which is defined as Eq. (25):

25 $${R}_{{\rm{T1}}}={R}_{{\rm{T0}}}+{R}_{{\rm{T0}}}{\alpha }_{{\rm{L}}}\Delta T$$

The thermal expansions ∆*A* and ∆*B* of the inner wall of the sleeve are defined as Eqs. (26) and (27), respectively:

26 $$\Delta A=\sqrt{\frac{[\pi {{R}_{T0}}^{2}{(1+{\alpha }_{L}\Delta T)}^{3}-\pi {{R}_{T0}}^{2}{(1+{\alpha }_{L}\Delta T)}^{2}{\alpha }_{E}\Delta T\tfrac{{L}_{T0}\rho g}{2{E}_{T0}}-(1+3{\alpha }_{L}\Delta T(1+{\alpha }_{L}\Delta T))(\pi {{R}_{T0}}^{2}-{A}_{T0}{B}_{T0})]{A}_{T0}}{(1+{\alpha }_{L}\Delta T-{\alpha }_{E}\Delta T\tfrac{{L}_{T0}\rho g}{2{E}_{T0}}){B}_{T0}}}-{A}_{T0}$$

27 $$\Delta B=\sqrt{\frac{[\pi {{R}_{T0}}^{2}{(1+{\alpha }_{L}\Delta T)}^{3}-\pi {{R}_{T0}}^{2}{(1+{\alpha }_{L}\Delta T)}^{2}{\alpha }_{E}\Delta T\tfrac{{L}_{T0}\rho g}{2{E}_{T0}}-(1+3{\alpha }_{L}\Delta T(1+{\alpha }_{L}\Delta T))(\pi {{R}_{T0}}^{2}-{A}_{T0}{B}_{T0})]{B}_{T0}}{(1+{\alpha }_{L}\Delta T-{\alpha }_{E}\Delta T\tfrac{{L}_{0}\rho g}{2{E}_{T0}}){A}_{T0}}}-{B}_{T0}$$

As shown in Fig. 4b, the size of the mold core at room temperature is *a*_T0_ × *b*_T0_, and its thickness is *l*_T0_. The height of the mold core after thermal deformation is *l*_T1_, which is defined by Eq. (28): The deformation caused by a change in the elastic modulus is denoted as ∆*l*_ET_, and the deformation caused by thermal expansion of the material is denoted as *∆l*_α_. The thermal expansion values of the mold cores, ∆*a* and ∆*b*, are defined by Eqs. (29) and (30), respectively:

28 $${l}_{{\rm{T1}}}={l}_{{\rm{T0}}}+\Delta {l}_{{\rm{ET}}}+\Delta {l}_{\alpha }={l}_{{\rm{T0}}}-{\alpha }_{{\rm{E}}}\Delta T\frac{{{l}_{{\rm{T0}}}}^{2}\rho g}{2{E}_{{\rm{T0}}}}+{l}_{{\rm{T0}}}{\alpha }_{{\rm{l}}}\Delta T$$

29 $$\Delta a=\sqrt{\frac{1+3{\alpha }_{{\rm{L}}}\Delta T(1+{\alpha }_{{\rm{L}}}\Delta T)}{1+{\alpha }_{{\rm{L}}}\Delta T-{\alpha }_{{\rm{E}}}\Delta T\tfrac{{l}_{{\rm{T0}}}\rho g}{2{E}_{{\rm{T0}}}}}}\,{a}_{{\rm{T0}}}-{a}_{{\rm{T0}}}$$

30 $$\Delta b=\sqrt{\frac{1+3{\alpha }_{{\rm{L}}}\Delta T(1+{\alpha }_{{\rm{L}}}\Delta T)}{1+{\alpha }_{{\rm{L}}}\Delta T-{\alpha }_{{\rm{E}}}\Delta T\tfrac{{l}_{{\rm{T0}}}\rho g}{2{E}_{{\rm{T0}}}}}}\,{b}_{{\rm{T0}}}-{b}_{{\rm{T0}}}$$

The designed gaps between the mold core and sleeve at the molding temperature are *k*_T1_ and *j*_T1_, which are defined as Eqs. (31) and (32), respectively:

31 $${k}_{{\rm{T1}}}={B}_{{\rm{T0}}}+\Delta B-{b}_{{\rm{T0}}}-\Delta b$$

32 $${j}_{{\rm{T1}}}={A}_{{\rm{T0}}}+\Delta A-{a}_{{\rm{T0}}}-\Delta a$$

### DSMLA alignment error control method

To ensure that the formed MLA is smoothly released from the mold, the thermal expansion coefficient of the glass material must be greater than that of the sleeve material when the system is below the *T*_g_, such that the glass lens does not squeeze the sleeve during cooling. The thermal expansion coefficient of the mold core material should also be greater than or equal to the thermal expansion coefficient of the sleeve.

Based on the above requirements, to predict and control the alignment error of the DSMLA, we develop a mold design with multiple materials. Different materials were used for the mold cores and sleeve to ensure that the mold cores achieved high surface accuracy during the large-area processing of the DSMLA. This process also ensured that the mold cores and sleeve would not be squeezed during molding, allowing the lens to be released.

Using this method, the designed alignment error of the DSMLA was used to calculate the theoretical gap between the mold cores and sleeve, and the sizes of the mold cores and sleeve at high temperatures were obtained. The room-temperature dimensions of the mold cores and sleeve were obtained by deriving their nonlinear thermal expansion to ultimately predict and control the alignment error of the DSMLA.

### DSMLA mold design

Next, we verify the control of the alignment error of the DSMLA molding by the multi-material combination of mold designs. A heat-resistant stainless steel substrate with a Ni-P plating was selected as the mold core material, and the sleeve comprised cemented carbide. The mold design met the requirements that the thermal expansion coefficient of the glass material be larger than that of the sleeve material, while the thermal expansion coefficient of the mold core material was larger than that of the sleeve. Therefore, this material combination meets the requirements of mold processing and use, and the mold material is not limited by this scheme. While the thermal expansion coefficient of each part of the mold satisfies the specified relationship and each material satisfies the requirements of mold processing and use, the mold can be manufactured using a variety of material combinations.

**Fig. 5:**
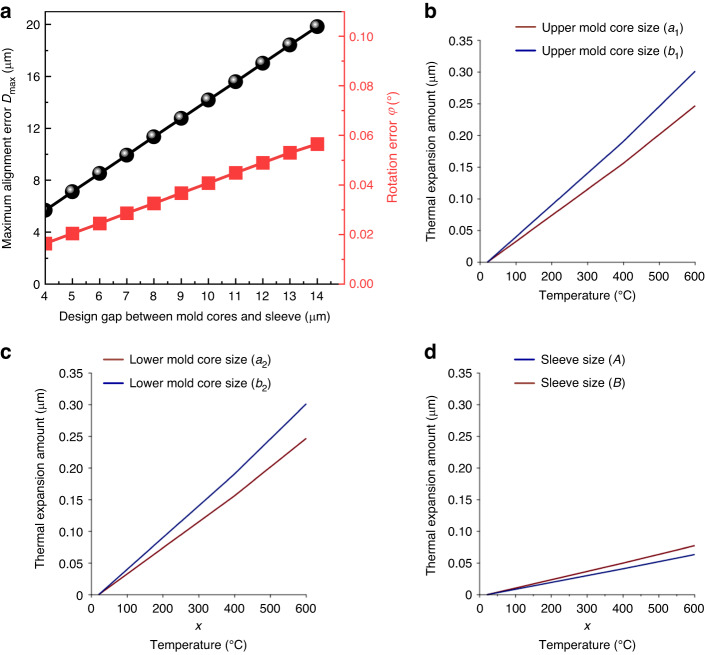
The prediction of the DSMLA maximum alignment error. **a** Maximum theoretical alignment error of the DSMLA. Thermal expansion of the mold assembly: **b** Calculated thermal expansion of the upper mold core. **c** Calculated thermal expansion of the lower mold core. **d** Calculated sleeve thermal expansion

The dimensions of the DSMLA were 23 mm × 28 mm × 8.3 mm. As shown in Fig. 5a, through the above thermal expansion calculation and alignment error calculation, the maximum theoretical alignment error of the DSMLA under different gaps in the sleeve and the mold cores at the molding temperature was predicted. The minimum gap between the mold cores and the sleeve was set to 4 μm (Table 1).

**Table 1:** Physical and thermomechanical properties of the materials

Item	Heat-resistant stainless steel	Cemented carbide	Glass (D-K59)
Coefficient of expansion *α* (10^−6^/°C)	18.09 (400 °C) 19.92 (600 °C)	4.7 (400 °C) 4.9 (600 °C)	6.3 (70 °C) 7.8 (300 °C)
Young’s modulus *E* (10^7^ Pa)	20,000	57,000	8318
Transition temperature *T*_g_ (°C)	——	——	497
Yield temperature *T*_S_ (°C)	——	——	551
Annealing temperature (°C)	——	——	460

Based on the predicted DSMLA alignment error, the mold cores and sleeve were designed and manufactured. The corresponding sizes are listed in Table 2. The material parameters and dimensions of the sleeve and mold cores were substituted into Eqs. (26), (27), (29), and (30) to obtain the size change curves of the sleeve and the mold cores at different temperatures. When the molding temperature was 575 °C, the maximum alignment error occurred when the relative rotation angle of MLA1 and MLA2 was 0.04° without considering the deflection of the DSMLA (MLA1 and MLA2 were parallel by default). The theoretical maximum alignment error of the lens unit on the DSMLA was 16.16 μm.

**Table 2:** Size of the mold cores and sleeve

Item	Size at 20 °C
Inner wall length of sleeve (*A*)	22.980 mm
Inner wall width of sleeve (*B*)	28.033 mm
Sleeve thickness (*L*)	20.232 mm
Outer wall radius of the sleeve (*R*)	23.460 mm
Upper mold core size (*a*_1_)	22.793 mm
Upper mold core size (*b*_1_)	27.806 mm
Lower mold core size (*a*_2_)	22.796 mm
Lower mold core size (*b*_2_)	27.808 mm

## Experiment and discussion

### Experimental

The sleeve and concave MLA mold cores were designed and manufactured according to the mold design method as described above. A Mitutoyo three-coordinate measuring instrument was used to measure the mold cores and cemented carbide sleeves. The PGM experiment was carried out on a custom high-precision molding machine, and the glass preform was a square glass (DK-9) provided by the Chengduguangming Company. The molding temperature was set to 575 °C, the molding pressure was set to 3600N, and a multi-step molding method was used^38^. The alignment error of the fabricated DSMLA was measured, and four groups of MLA units were taken on both sides of the DSMLA. The peak-to-valley (PV) and surface roughness (Ra) of the microlens unit were measured by a tactile method (Taylor Hobson PGI). The alignment error of the DSMLA was measured by a center deviation measuring instrument (OptiCentric). First, the distance of the curvature center of the MLA1 and MLA2 units in the vertical direction was measured. Then, the rotation error, shift error, and maximum alignment error of the DSMLA were calculated based on the positional relationship of each group of microlens units.

### Results and discussion

#### Surface geometry measurements

**Fig. 6:**
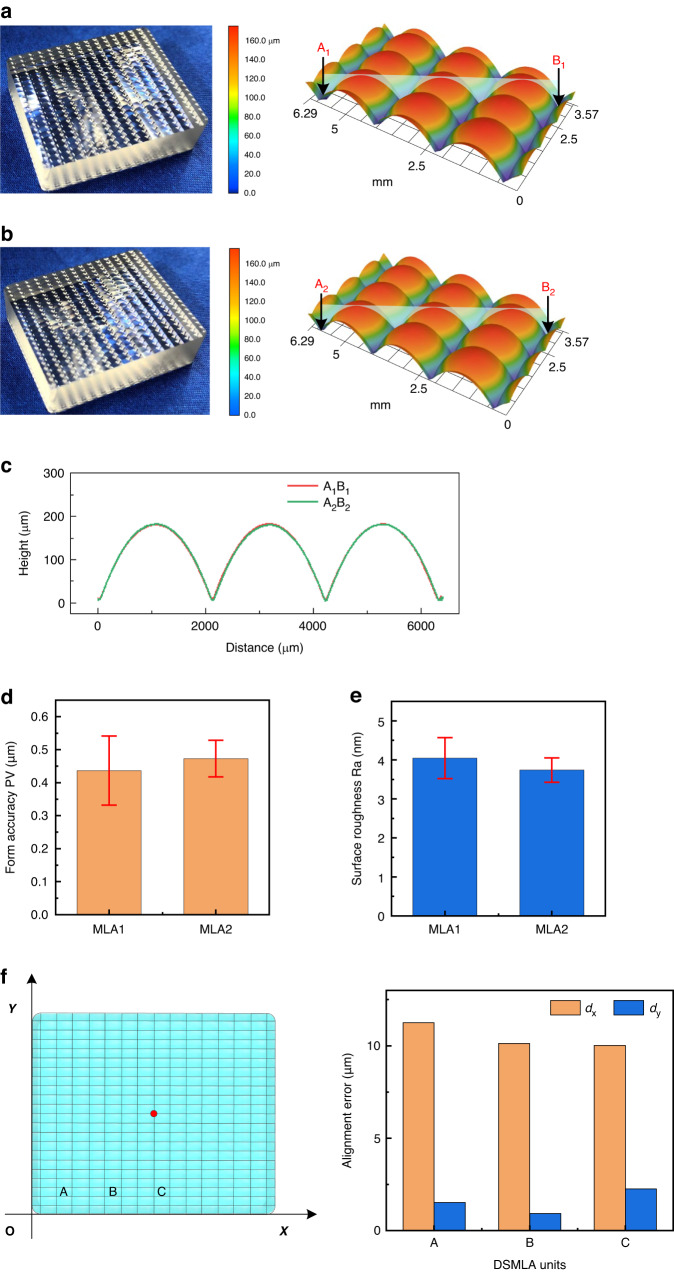
Molded DSMLAs. **a** MLA1, **b** MLA2, and **c** contours of MLA1 and MLA2. **d**, **e** Form accuracy and surface roughness of the microlens array units of the DSMLA. **f** Alignment error measurements of the DSMLA

The molded DSMLA is shown in Fig. 6a, b, and the overall dimensions of the MLA were 23 mm × 28 mm × 8.3 mm. Each side comprised 20 × 12 rectangular adjacent convex lenses to form an MLA. The size of each lens unit was set at 1 mm × 1.8 mm, the radius of curvature was 3.1 mm, and the MLAs on both sides of the lens were identical. According to the laser confocal microscopy measurements, the MLA1 and MLA2 profiles were highly consistent. The profilometry measurements showed that the average PV value of MLA1 was 0.44 μm, and the average PV value of MLA2 was 0.47 μm. The average Ra of MLA1 was 4.04 nm, and that of MLA2 was 3.74 nm. These measurements indicate that the DSMLA exhibited an extremely high surface quality.

The alignment errors of the DSMLA are shown in Fig. 6f. The results are the offset errors of the three sampling units (A, B, and C) on the DSMLA in the *x-* and *y*-directions. Calculations showed that the shift error of the DSMLA was 10.59 μm, the rotation angle was 0°, and the maximum alignment error was 10.59 μm. The experimental results showed that the actual alignment error of the DSMLA was 5.57 μm less than the theoretical maximum alignment error, and the actual rotation angle was 0.04° less than the theoretical maximum.

The experimental results were in the range of the theoretical predictions, which verified the efficacy of the theoretical design approach. However, it was very difficult to achieve the theoretical maximum error because the positions of the upper and lower mold cores in the sleeve changed randomly and were affected by the initial assembly position (which occurred at room temperature). Therefore, the alignment error of manufactured DSMLAs was generally less than the theoretical maximum value, and this value could not be accurately predicted.

In a follow-up study, it will be valuable to consider errors in the measured sizes of the mold cores and sleeve should be accounted for. In addition, when measuring the alignment error of the DSMLA, we set the optical axis of MLA1 and the optical axis of MLA2 to be parallel to each other, but due to the manufacturing error of the mold cores and the sleeve, these optical axes were not parallel, which increased the alignment error. The experimental results showed that the mold designed by a combination of multiple materials allowed the DSMLA to be released smoothly, helping control the alignment error.

#### Optical performance measurements

**Fig. 7:**
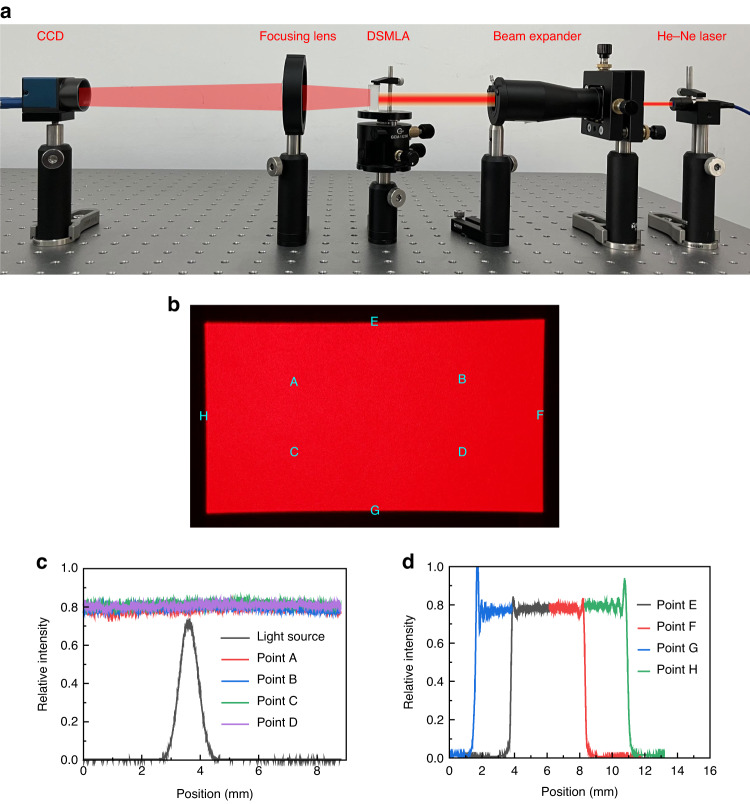
The measurements of the beam homogenization performance of the DSMLA. **a** The setup of the optical system of the DSMLA beam homogenizer. **b** Homogenized beam spot, **c** measurement results of beam spot uniformity, and **d** measurement results of beam spot sharpness

To measure the actual beam homogenization performance of the DSMLA, a He-Ne laser with a wavelength of 632.8 nm and a Gaussian intensity distribution was selected as the light source, and a CCD (MER2-2000-19U3M) was used to collect the beam spot. The laser homogenization test optical path shown in Fig. 7a was constructed. The pixel size of the CCD was 2.4 μm × 2.4 μm, and the resolution was 5496 (*H*) × 3672 (*V*). As shown in Fig. 7b, the output beam spot shaped by the molded DSMLA was close to the flat-top shape, and the spot size was 26.86 mm × 49.96 mm. As shown in Fig. 7c, all four positions on the spot were evenly spaced to measure the uniformity of the sample. As shown in Fig. 7d, the edge sharpness was measured on four sides of the spot. The image collected by the CCD was processed to obtain the gray value of each pixel, and the average gray value of all pixels was calculated according to Eq. (33), where *n* is the total number of all pixels, *Ф*_i_ is the gray value corresponding to the pixel point, and *γ* is the uniformity, which is defined by Eq. (34)^39,40^.

The spot uniformity *γ* was calculated to be 97.81%, and the effective homogenization area accounted for 91.66% of the total area. The experiment showed that the DSMLA homogenized the Gaussian laser:

33 $$\bar{\phi }=\frac{{\sum }_{i=1}^{n}{\phi }_{{\rm{i}}}}{n}$$

34 $$\gamma =1-\frac{1}{\bar{\phi }}\cdot \sqrt{\frac{{\sum }_{i=1}^{n}{({\phi }_{i}-\bar{\phi })}^{2}}{n}}$$

## Conclusion

In this paper, a novel multi-material combination of mold assemblies was investigated to improve the alignment accuracy of the DSMLA in PGM. The influence of the DSMLA alignment error on the beam homogenization performance was analyzed, and a set of equations were established to predict and control the alignment error. The accuracy of these equations was verified via PGM experiments. The following conclusions were drawn:

(1) The shift error and rotation error of the DSMLA decreased the beam homogenization capacity. This shift error reduced the uniformity of the spot, and when it exceeded half the size of the lens unit, the spot morphology changed abruptly. The rotation error caused the uniform area of the spot to decrease, the overall area of the spot to increase, and the sharpness of the spot to decrease.

(2) The alignment error of the DSMLA was mainly determined by the size of the gap between the mold cores and the sleeve at the molding temperature. A method to optimize this gap size based on the thermal expansion difference between the mold core and the sleeve was reported. The experimental results showed that the corresponding mold design reduced the alignment error of the DSMLA.

(3) The DSMLA molded by combining molds with different materials exhibited an extremely high manufacturing accuracy and consistency. The experimental results showed that the uniformity of the homogenized spot of a Gaussian-distributed laser beam reached 97.81%, and the effective uniform beam spot area accounted for 91.66% of the total.
